# Evaluation of the Influence of Surface Roughness Parameters on Ultrasonic Rayleigh Waveforms

**DOI:** 10.3390/ma17225493

**Published:** 2024-11-11

**Authors:** Karol Grochalski, Jakub Kowalczyk, Marian Jósko, Michal Wieczorowski

**Affiliations:** 1 Faculty of Mechanical Engineering, Institute of Mechanical Technology, Poznan University of Technology, 60-965 Poznan, Poland; karol.grochalski@put.poznan.pl; 2 Faculty of Civil and Transport Engineering, Institute of Machines and Motor Vehicles, Poznan University of Technology, 60-965 Poznan, Poland; jakub.kowalczyk@put.poznan.pl (J.K.); marian.josko@put.poznan.pl (M.J.)

**Keywords:** non-destructive testing, ultrasonic testing, surface wave, surface conditions

## Abstract

Ultrasonic nondestructive testing is widely used not only in the laboratory, but also in industry. The tests use various types of ultrasonic waves, diverse measurement techniques and different apparatus. One of the problems encountered is the high susceptibility of the surface wave to interference. Some of the interference is random in nature and can be minimized (e.g., contamination of the surface or resting a finger on the surface under study). Some of the interference is permanent in nature, such as variable surface roughness. The aim of the conducted research was to evaluate the influence of roughness on ultrasonic wave propagation. The study used samples with surface roughness Sa from 0.28 to 219 µm, and ultrasonic surface wave probes with frequencies from 1.41 to 8.02 MHz. It was observed that roughness significantly affects the attenuation of the ultrasonic wave, and the differences in signal amplification reached more than 15 dB. Similarly, the effect of the ultrasonic wave’s transit time through surfaces of different roughness was noted. It was found that the difference in the ultrasonic wave transition time was more than 50 µs. The results of the study can be helpful for the ultrasonic testing of materials with different surface conditions.

## 1. Introduction

Modern industry requires quality control of manufactured components. Various inspection methods are used in quality testing. The main division is between destructive and non-destructive methods. Non-destructive methods include techniques such as visual, penetrant, eddy current, ultrasonic and X-ray-based methods. Each of these methods has its advantages and limitations. The methods that allow examination of the interior of objects are ultrasonic and X-ray-based techniques. X-ray-based methods enable efficient examination of objects both in production and laboratory conditions [1]. For example, in [2], research was conducted using X-ray microtomography and ptychography, where the authors used these methods to characterize the three-dimensional lattice structure, morphology and distribution of metal carbides in the as-cast nickel superalloy IN713LC. These findings demonstrate the high sensitivity and accuracy of these methods. The ultrasonic method under study does not offer such high resolution, but it is significantly faster and cheaper. Moreover, the resolution achieved is sufficient for industrial applications and specific laboratory work. A non-destructive method that offers great insight is the ultrasonic method. This method uses ultrasonic wave transition, reflection and refraction phenomena. It makes it possible to detect surface and subsurface cracks [3,4] and evaluate welded joints [5,6] and diffusion joints [7]. Ultrasonic testing commonly uses longitudinal waves and transverse waves [8].

It has been noted that it is possible to make greater use of Rayleigh waves in ultrasonic testing than before. An ultrasonic Rayleigh wave is a wave that propagates along the surface of the elements under study, penetrating to a small depth. Although the first description of surface waves was presented by Lord Rayleigh in 1885, the problem of surface waves is still relevant, as evidenced by the wide range of ongoing research in the area of these waves [9,10]. Research in the area of ultrasonic Rayleigh waves covers a wide range. Part of the work conducted included non-contact laser ultrasonic methods for studying the propagation of Surface Acoustic Wave (SAW) on curved surfaces. The authors [11] have shown that they eliminate coupling issues in wave generation and detection, and their high temporal resolution makes it possible to study the dispersion effect over a large frequency range. In the work [12], it was shown that it is possible to detect cracks of very small depth (h ≈ 80 μm) compared to the Rayleigh wavelength (λ ≅ 2 mm). In this work, the Rayleigh wave was used to study fatigue cracking. The results confirmed that macro-cracks lead to a significant decrease in the transmission of the Rayleigh wave. In addition, it was found that the acoustic nonlinearity parameters initially stabilize, after which they significantly increase from the pristine state to the propagation of microcracks. The obtained index can be used for localization and characterization of fatigue damage. The authors confirmed that the study of the multicomponent nonlinear interaction between Rayleigh waves and evolving fatigue damage can provide clues to nonlinear Rayleigh wave detection of evolving fatigue damage.

The temporal resolution makes it possible to study the dispersion effect over a large frequency range. There is work being done showing that surface wave dispersion measurements can be used to nondestructively characterize shot-peened, laser shock-peened, burnished and otherwise surface-treated specimens [13]. The results suggest that a diffraction correction may be introduced to increase the accuracy of surface wave dispersion measurements. A simple diffraction correction model was developed for surface waves and this correction was subsequently validated by laser-interferometric velocity measurements on aluminum specimens. However, the work carried out covered a relatively small range of variation in surface parameters. Work has also been carried out in which a theoretical model, along with experimental verification, is developed to describe the generation, propagation and reception of a Rayleigh wave using angle beam wedge transducers [14,15]. One paper [16] proposed a geometry model for determining Rayleigh wave beams using an area sound source in the x–y plane. The results obtained confirmed that the oscillatory motion for Rayleigh waves is elliptical in nature. In addition, it was confirmed that the distributions of received waves at different propagation distances depend not only on the size of the receiver, but also on the attenuation of the Rayleigh wave. The authors performed simulations and proposed their own model. The results obtained using the proposed model and three other simple models were compared. The resulting differences between these results were discussed in detail. The results confirmed an effective tool for nondestructive testing and evaluation of Rayleigh waves using wedge beam transducers under practical conditions.

Additional research is being conducted, including ultrasonic studies utilizing surface waves. In reference [17], the authors employed Rayleigh-like surface waves, generated and detected in a pitch-catch configuration, to mitigate the effects of masking. By using electromagnetic acoustic transducers (EMATs) with a minimal standoff, it is possible to surpass many of the current speed limitations in rail testing, achieving high accuracy in detecting and assessing surface defects. The article presents experiments on rail samples with both real and machined defects, where EMATs were used to generate and detect low-frequency wide-band surface waves. The depth of these defects can be characterized by analyzing both the amplitude of the time-domain signal and the frequency-dependent behavior.

Paper [18] used an ultrasonic method to detect cracks during operation (on-line). The authors noted that most of the previous studies involved off-line testing. In this work, the authors also used a surface wave and showed that a suitable candidate is the Surface Acoustic Wave approach (SAW). The apparatus was shown to be quite compact, and it was also found that the use of high-frequency transducers allows for high resolution while being robust with respect to environmental noise.

Ultrasonic Rayleigh waves have been used in tests on a variety of materials, including composite materials [19] and on metals [20]. Tests have been conducted in which fatigue cracks have been detected as they form.

Ultrasonic surface waves have also been used to study the shear-thickness of a path applied to steel profiles [21] and to study the kinetics of two-way adhesive bonding [22]. There has also been work on how in situ monitoring of stresses provides a crucial input for residual life prognoses and is an integral part of structural health monitoring systems [23].

Analyzing the available literature, no results were encountered on the influence of post-surface conditioning on Rayleigh wave properties.

The primary objective of the research is to find out the influence of roughness parameters on the propagation of the surface wave, the Rayleigh wave. The analysis of selected parameters in the context of wave propagation should allow us to understand the relationship between the parameters of the ultrasonic wave and the surface structure of the components under study. The research also aims to identify potential practical applications in ultrasonic research using surface waves.

## 2. Research

### 2.1. Research Procedure

**Figure 1:**
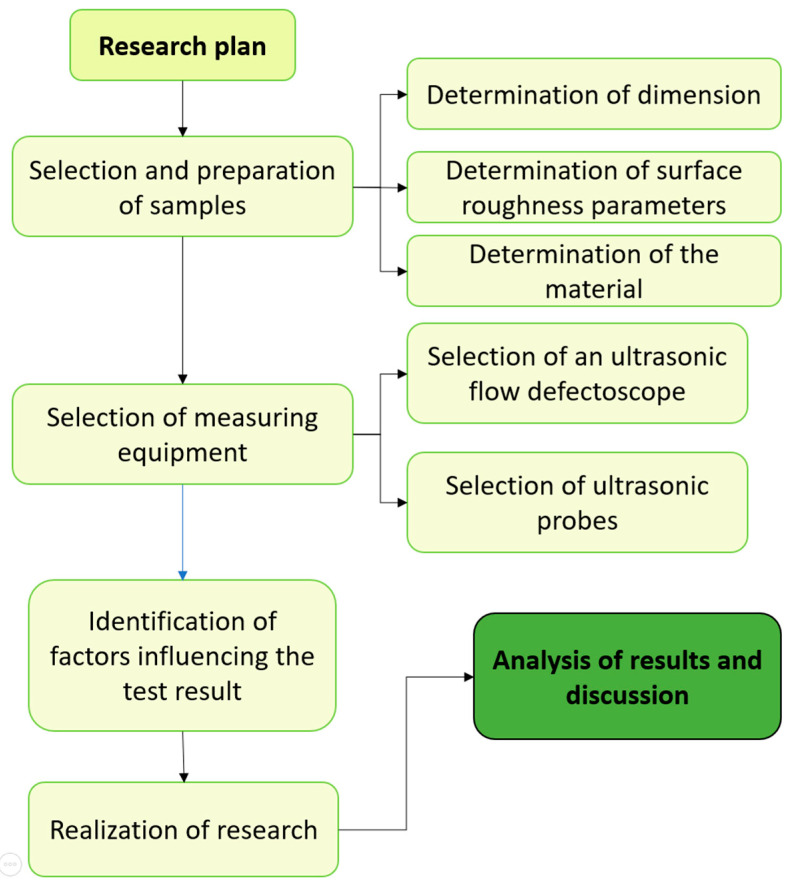
Research plan.

The experiment consisted of studying the passage of an ultrasonic Rayleigh wave through surfaces with significantly different surface roughness parameters. A digital ultrasonic defectoscope was used, and anything that influenced the test results was investigated and kept to a minimum. Measurement errors were determined for the measurement systems used. All research work was carried out based on the test procedure shown in Figure 1.

The research began with the selection of measurement samples. As part of this step, the dimensions of the specimens, the materials from which they were made and the roughness parameters were determined. The samples were then fabricated using a digitally controlled machining center (CNC). In the next step, ultrasonic heads and an ultrasonic defectoscope were selected from digital industrial apparatuses. Once the apparatus was chosen, the factors that could affect the test results were identified. Subsequently, the surface roughness parameters were verified [24], and the testing system was prepared to minimize the influence of factors affecting the test results. Finally, measurement errors and ultrasonic wave velocities were determined, allowing for testing and analysis of the results.

### 2.2. Materials and Methods

In a production environment, ultrasonic testing is usually carried out using ultrasonic testing probes with frequencies of between 2 and 10 MHz, and most objects that are tested using ultrasonic methods are made of steel. There are special cases where higher-frequency waves (up to about 20 MHz) are used, but these are limited to testing welded joints or adhesive joints. When selecting ultrasonic probes for testing, the depth of penetration of the Rayleigh wave is also important. This frequency–depth pair is, for the selected transducers and the tested material 1.44 MHz–0.33 mm, for 4.22 MHz–0.11 mm and for a frequency of 8.02 MHz–0.06 mm. The wave frequency and roughness parameters selected and considered in the research are the most frequently used in industrial conditions and therefore have the greatest practical importance. For this reason, the test specimens were made from 18G2A steel, a high-strength, weldable steel. The chemical composition is shown in Table 1.

**Figure 2:**
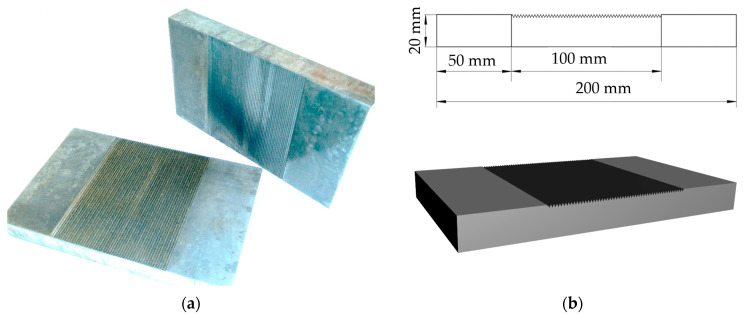
Sample used in the study; (**a**) general view; (**b**) dimensions.

The softened hardness of 18G2A steel is 220 HB, the tensile strength Rm is 490–630 MPa and the yield strength Re is 335 MPa. A drawing of an example specimen is shown in Figure 2a and its dimensions as well as model are shown in Figure 2b.

Steel is widely used, mainly for building structures and bridges, and it was decided when planning the specimens to reflect real conditions as closely as possible. Therefore, the thickness of the specimen was assumed to be 20 mm, the area of the field with the roughness was 100 × 120 mm and the whole specimen was 200 mm, i.e., a field of 50 mm was left—the place where ultrasonic probes are applied, according to Figure 2b.

**Figure 3:**
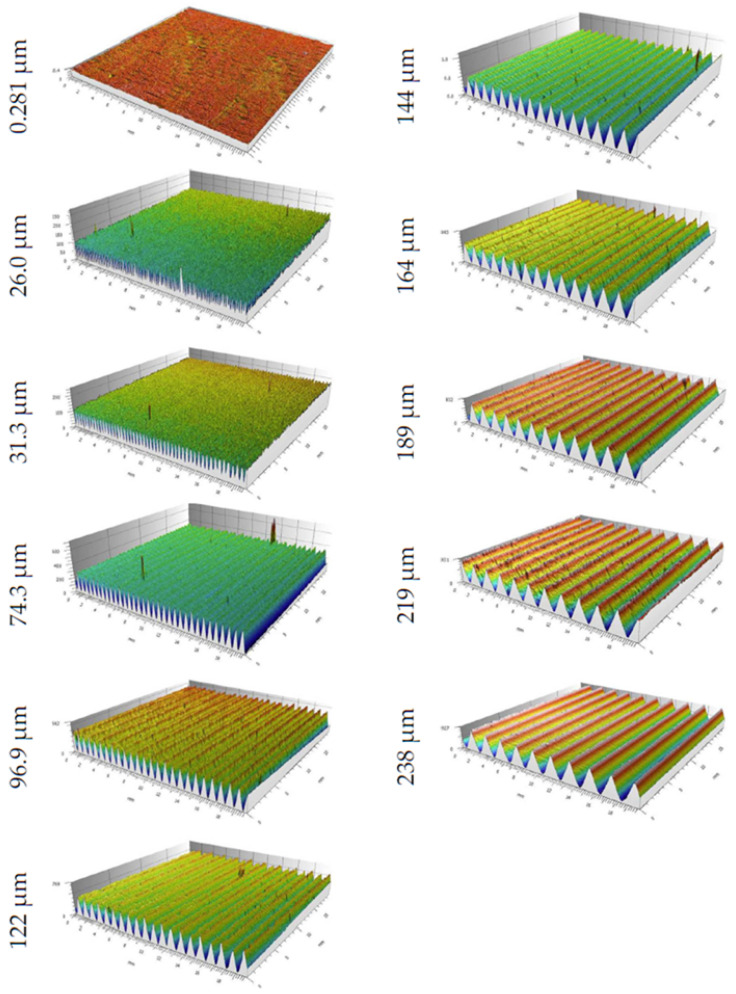
Surface parameters of the samples used.

**Figure 4:**
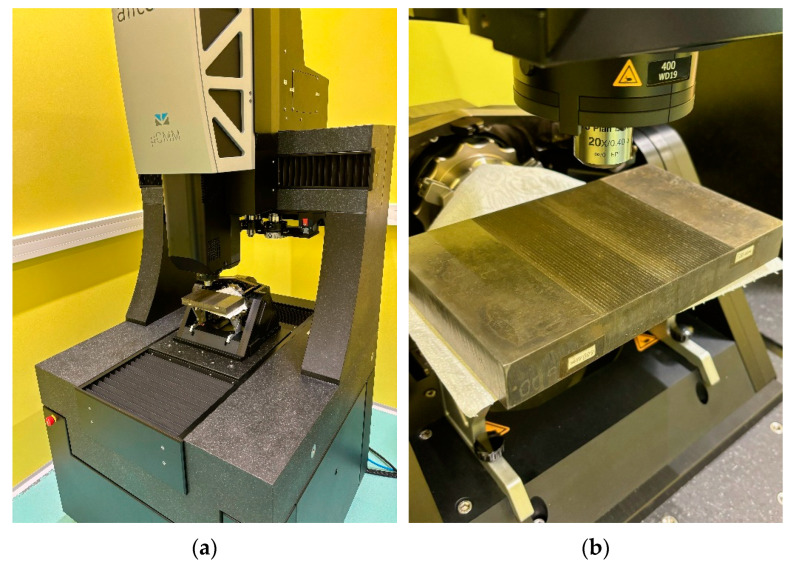
Measurements of the surface parameters of the test samples; (**a**) view of station, (**b**) view of measuring head.

Bearing in mind the differences between optical and tactile methods of surface topography measurements [25] and considering the speed of measurement, an optical technique was chosen to investigate surface asperities. The surface parameters were determined using the µCMM Alicona (operating on the focus variation technique, Graz, Austria), and the results of these measurements are shown in Figure 3. A view of the stand where the surface parameters were measured is displayed in Figure 4.

Focus Variation microscopy (FV) provides the best results for the analyzed surfaces because it is based on the texture present in the bright-field image. This technique is generally suitable for low magnification, where capturing the geometric macrostructure of the surface is most important, and where the use of other measurement methods, particularly contact profilometry, is not feasible due to the risk of damaging the measurement probe. In this technique, it is crucial to use proper lighting [26] and maintain consistent measurement conditions for each sample to avoid potential errors [27].

In the parametric analysis of the data, measurement segments appropriate for the periodic nature of the surface were individually selected based on the measured structure. The focus was primarily on the surface amplitude parameters. The obtained data were filtered (post-processed) to remove maximum amplitude deviations and measurement noise in order to accurately represent the characteristics of the studied structure.

In the study, a 20× magnification objective was used, providing a large measurement area, which is particularly important for amplitude changes on the surface. Due to the periodic nature of the texture, the Lateral Resolution parameters were defined at 0.5 µm, and the Vertical Resolution at 0.3 µm. The lighting parameters were set to High Dynamic Range mode, using coaxial illumination (Gain = 1, contrast = 0.54).

### 2.3. Ultrasonic Apparatus

**Figure 5:**
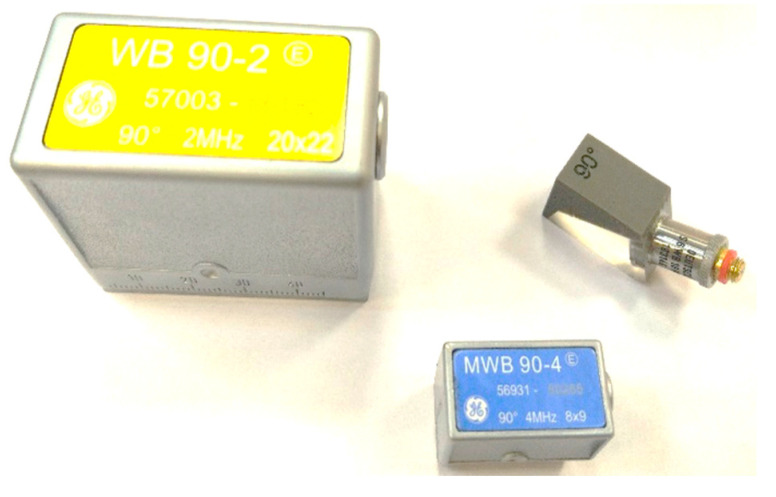
Ultrasonic probes used in research.

Due to the practical nature of the research, an industrial digital ultrasonic defectoscope USM35XS (General Electric Company, Boston, MA, USA) was used for the measurements, along with a set of ultrasonic probes. Three pairs of ultrasonic transducers were used, each with the same frequencies as those used for testing in real industrial environments. It was decided to choose the following probes: Karl Deutsch S6WB10WM (Karl Deutsch, Wuppertal, Germany), General Electric Company MWB 90-4 8x9 (General Electric Company, Boston, MA, USA) and General Electric Company WB 90-2 20x22 (Figure 5).

The actual frequency of the ultrasonic transducers used was checked prior to testing. A summary of the frequency given by the probe manufacturer and the measured frequency, as well as the dimensions of the transducers, are included in Table 2.

The next step was to study the factors likely to influence the test results. In this study, the ultrasonic transmission technique was used. This is a method in which the transmitting probe emits an ultrasonic wave that propagates across the surface of the test piece and reaches the receiving probe, and the only parameter that can be measured is the height of the pulse. With this method, a single pulse is generated on the defectoscope screen.

Measurement results can be influenced by factors such as the angle of the ultrasonic probes to each other, the distance between the ultrasonic probes, the condition of the coupling medium, contamination of the test surfaces and the configuration of the defectoscope.

**Figure 6:**
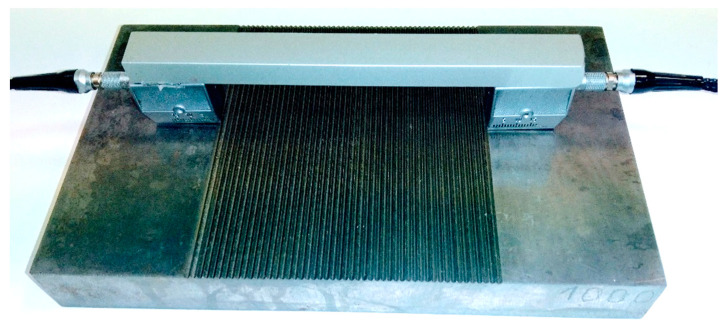
Ultrasonic probes used in research.

To reduce the influence of these factors on the test results, the ultrasonic probes were bonded to an aluminum profile. This mounting of the probes (Figure 6) guarantees a constant angle between the probes and a constant distance between them. In addition, the sample was degreased immediately prior to measurement and the Karl Deutsch EchoFluid coupling agent was applied between the probes and the surface, after which the probes were aligned parallel to the long edge of the sample and pressed with a constant force.

**Figure 7:**
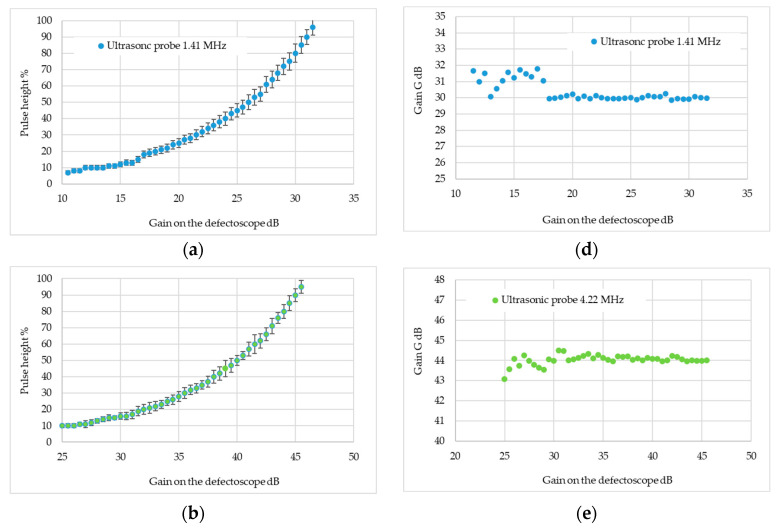

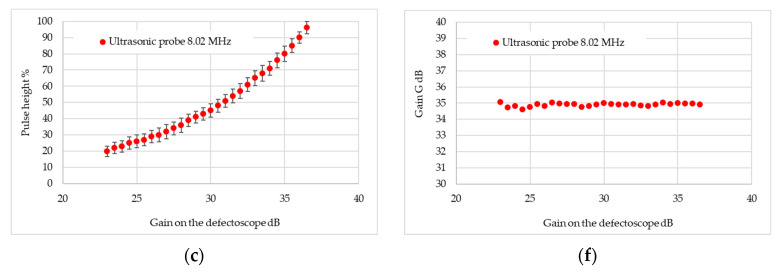
Testing the influence of amplification on the measurement result; Testing the influence of amplification on the measurement result; (**a**) change in pulse height depending on gain for an ultrasonic wave frequency of 1.41 MHz (graph includes error bars); (**b**) change in pulse height depending on gain for an ultrasonic wave frequency of 4.22 MHz (graph includes error bars); (**c**) change in pulse height depending on gain for an ultrasonic wave frequency of 8.02 MHz (graph includes error bars), analytically determined gain value for a pulse height of 80% of screen height at frequencies (**d**) 1.41 MHz, (**e**) 4.22 MHz and (**f**) 8.02 MHz.

The extent to which the defectoscope’s settings affect the measurement results was also checked; measurements were made for the probe with the highest and lowest frequencies. The pulse height on the defectoscope screen was measured by varying the gain of the defectoscope. The gain was changed so that the pulse height ranged from 10 to 100 per cent of the defectoscope screen height, then the analytical gain was determined for a pulse height of 80 per cent of the screen height. Each measurement was repeated ten times and the results are shown in Figure 7.

**Figure 8:**
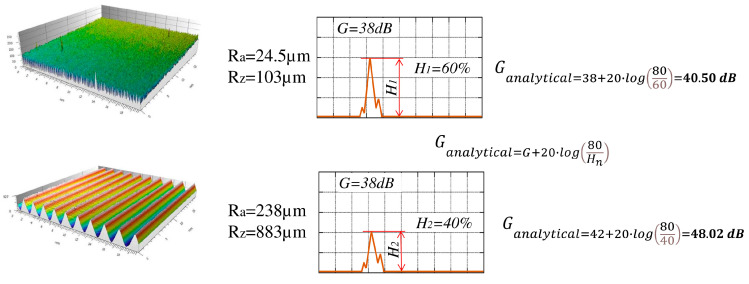
Study of the effect of gain on the measurement result.

Since the intended specimens had varying surfaces, it was envisaged that the tests would be carried out at a different gain, and the measure of the surface condition would not be the height of the pulse on the defectoscope screen but the gain value needed to obtain a pulse of 80 per cent of the screen. This gain value is denoted as G_analitical_. The dependence of the gain needed to obtain a pulse height of 80 per cent of the screen on the defectoscope gain is shown in Figure 8.

The graphs show that the least effect of the gain set on the defectoscope is observed for gains of more than 18 dB for the 1.41 MHz probe and 26.5 dB for the 4.42 MHz probe—it was decided that the tests would be performed for the indicated gains. For the 8.02 MHz head for the entire range of gains, no effect of gain on the measurement results was noticed.

In the next step, the ultrasonic Rayleigh wave velocity was determined for all the probes used, which allowed calibration of the ultrasonic equipment.

**Figure 9:**
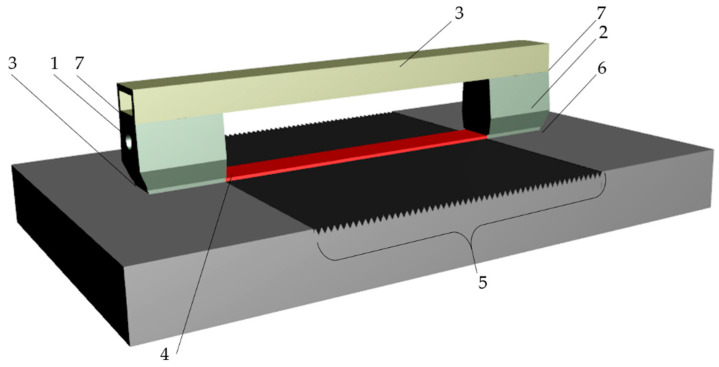
Measurement stand; 1—ultrasonic transmitting probe; 2—ultrasonic receiving probe; 3—cantilever; 4—Rayleigh wave; 5—tested surface; 6—coupling medium; 7—adhesive.

The final step in the study was to perform measurements for all samples and probes, during which the pulse heights on the defectoscope screen were recorded, as was the time of passage of the ultrasonic wave through the tested surface, according to the scheme shown in Figure 9.

The results of the research can be used in industrial conditions in two important areas. First, it is possible to assess the condition of a surface that is built up where it is not possible to use classical methods of assessing surface conditions. The second important area is that it is possible to make corrections to studies conducted using Rayleigh waves. One of the areas in which such waves are used in practice is the study of the quality of permanent joints, such as adhesive joints. Knowing the condition of the surface as a result of the machining method used, appropriate corrections can be made to assess the quality of the joint, and this has practical applications.

## 3. Results

**Figure 10:**
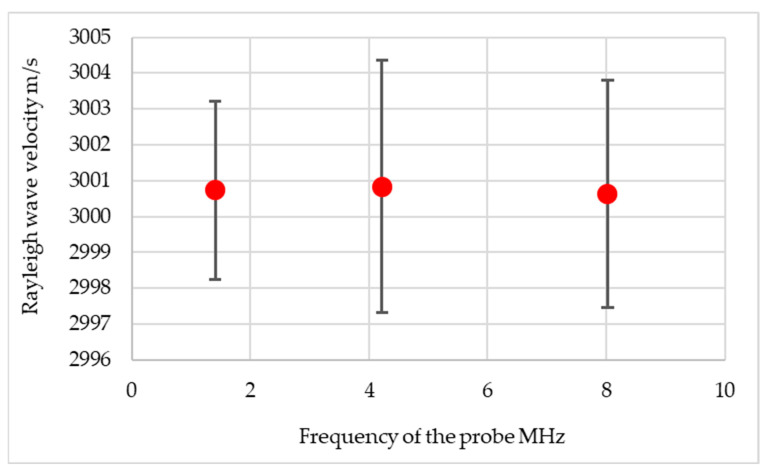
Rayleigh wave velocity.

The first stage of the research was to determine the speed of the ultrasonic wave. It was confirmed that the velocity of the ultrasonic wave does not depend on the frequency of the wave. The determined Rayleigh wave velocity was 3001 m/s, with detailed results shown in Figure 10.

As an ultrasonic measure, it is not the pulse height as implemented in classical testing but the gain of the defectoscope that allows the pulse height on the defectoscope screen to be 80 percent of the height of the entire screen. This is due to the relatively large influence of roughness on pulse heights and the need to conduct tests at different gains.

**Figure 11:**
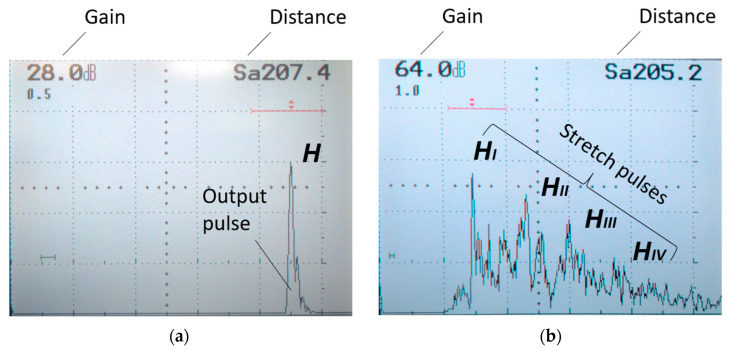
Marking the pulses on the defectoscope screen. (**a**) view for output pulse (**b**) view for a stretched pulse.

It is noted that higher roughness is followed by pulse stretching, allowing several partial pulses to be distinguished, labeled, for testing, H_I_, H_II_, H_III_ and H_IV_ (Figure 11).

The results of the pretreatment measurements are shown in Table 3, Table 4 and Table 5.

**Figure 12:**
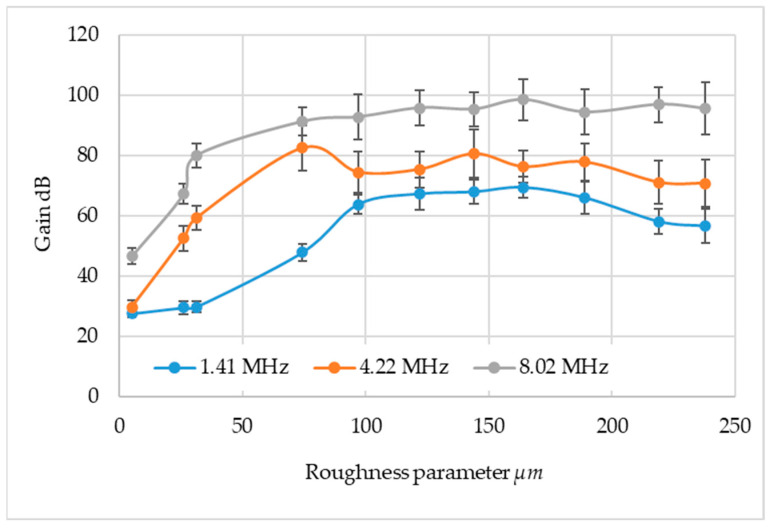
Research results in the area of gain.

Conducting an analysis of the results, defectoscope gains were determined such that the pulse height was 80 percent of the screen. These results are shown in Figure 12.

For further analysis, the ultrasonic wavelength was determined by dividing the wave speed by its frequency. For the frequency of 1.41 MHz, the wavelength was 2.13 mm, for 4.22 MHz it was 0.71 mm, while for 8.02 MHz the wavelength was 0.374 mm.

**Figure 13:**
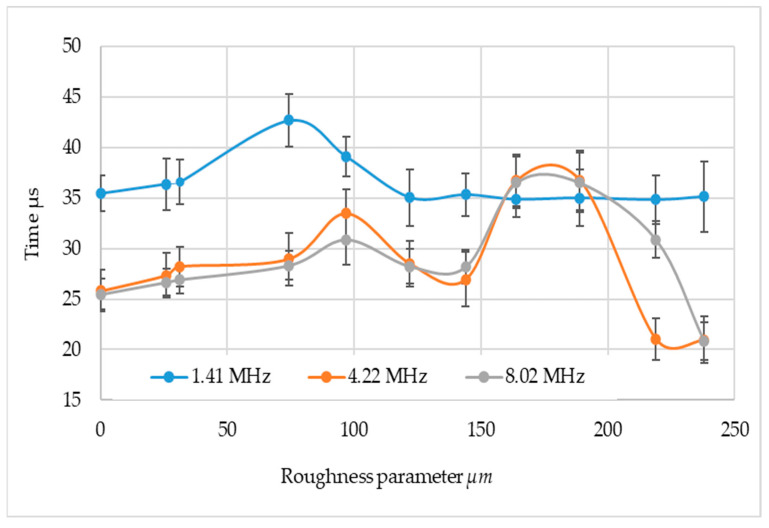
Research results in the area of time wave transitions.

During the measurements, the ultrasonic wave transit time was also recorded for all measurement systems. Since the probes were of different sizes, these times differ from each other. What is important, however, is their change with the change in the state of the surface of the tested materials. The aggregate results are shown in Figure 13.

Analyzing the results in the gain area, it was found that all three frequencies show a certain pattern of gain increase as the area parameter increases up to a certain point, followed by a decrease. A similar character pattern of gain for all frequencies used was observed for Sa in the range of 0.281 to 75 µm—a gain increase of about 20%. Then, for Sa in the range of 75 to 165 µm, there is a slow increase for ultrasonic probes at frequencies of 1.41 MHz and 8.02 MHz. For the frequency of 4.22 MHz, a slight gain waver is noted. For the 1.41 and 4.22 MHz frequencies, a decrease in gain was noted at the highest waviness (above 164 µm). For the frequency of 8.02, a gain stabilization was noted at this roughness. The frequency of 1.41 MHz shows the greatest variation and highest gain values, which is due to the fact that the attenuation coefficient increases with increasing frequency; 4.22 MHz and 8.02 MHz have more similar values and similar shapes of curves.

The 8.02 MHz wave is the smallest wavelength, it is the most attenuated by the structure of the steel material and scattered by roughness, but at the same time the test system has a high defect susceptibility.

Analyzing the passage time of an ultrasonic wave through an area with different surface parameters, it can be seen that the least susceptible to the surface condition is the wave with a frequency of 1.41 µm. This is probably due to the fact that it is the longest wavelength wave, at the same time it has the lowest testing resolution.

A similar character of changes was noticed for higher frequencies. For all frequencies, an increase in the transition time of the ultrasonic wave was found for Sa below 75 µm. Then, for frequencies 4.22 and 8.02 MHz, a significant increase in this time was noted from about 27 µs (at Sa equal to 144 µm) to about 37 µs at Sa = 164 and Sa = 189 µm. Subsequently, it was noted that the ultrasonic wave transition time was significantly reduced by about 40%, or to 21 µs. It was noted that the highest roughness and high frequency ultrasonic wave transition time is shorter than when the ultrasonic wave passes over a surface with low Sa—0.281 µm. The way of mounting the ultrasonic probes (fixed connection) to the profile excludes the change of the distance between them; it can be assumed that there was a transposition of the ultrasonic wave from a Rayleigh wave to a Stoneley wave.

## 4. Conclusions

The results confirmed that the condition of the surface affects the ultrasonic Rayleigh waveform. Changes were observed in three areas:

- The time it takes for the wave to pass through a particular section of the surface.
- The change in pulse height on the screen of the ultrasonic defectoscope.
- The change in pulse shape—including the appearance of additional pulses resulting from the transposition of the ultrasonic wave.

It was also found that the changes in the signal on the screen of the defectoscope depend significantly on the ultrasonic wavelength. The wavelength is dependent on the frequency of the ultrasonic probes and the speed of the wave. The research has also shown that it is worthwhile to determine the actual frequency of the ultrasonic probe before starting the measurements, as it can differ significantly from the frequency quoted by the probe manufacturer.

The results of the research, although carried out in the laboratory, can be used in industrial applications. It is possible to assess the condition of the surface to be tested using the surface wave. This is particularly important in those facilities where the test area is built up and it is not possible to classically assess the condition of its surface.

The results obtained in this work allow practitioners in industrial non-destructive testing to take into account specific corrections to the amplification/gain of the surface wave pulse and the selection of its frequency, resulting from the roughness of the ultrasonic tested surfaces. On the other hand, the planned investigations using the spectral analysis of the ultrasonic surface wave pulse will allow for a more accurate interpretation, especially of pulses stretched in time, caused by wave scattering on roughness and structural dissipation of wave energy. However, the amplitude–frequency analysis of ultrasonic wave pulses requires a separate research cycle.

## Figures and Tables

**Table 1:** Characteristics of the 18G2A steel used in the study.

Designation	Numerical Classification	C [%]	Si [%]	Mn [%]	Cr [%]	Al [%]	Ni [%]	Cu [%]	S [%]	P [%]
18G2A	1.0562	<20	0.20–0.50	<0.015	<0.30	<0.02	<0.30	<0.30	<0.04	<0.04

**Table 2:** Properties of the ultrasonic probes used.

Designations	Manufacturer	Name	Transducer Dimensions	Declared Frequency	Actual Frequency
P1	Karl Deutsch	S6WB10WM	diameter 6 mm	10 MHz	8.02 MHz
P2	General Electric Company	MWB 90-4	8 × 9 mm	4 MHz	4.22 MHz
P3	General Electric Company	WB 90-2	20 × 22 mm	2 MHz	1.41 MHz

**Table 3:** Measurement results for the 1.44 MHz ultrasonic transducer.

Sa µm	Gain dB	H_I_ %	H_II_ %	H_III_ %	H_IV_ %
0.28	27	76			
26	31	96			
31.3	31	93			
74.3	45	58			
96.9	60	52			
122	64	55	46	37	
144	64	51	45	37	30
164	64	43	91	75	55
189	64	64	92	72	84
219	59	89	48	56	53

**Table 4:** Measurement results for the 4.22 MHz ultrasonic transducer.

Sa µm	Gain dB	H_I_ %	H_II_ %
0.28	30	83	
26	48	48	
31.3	55	49	
74.3	70	19	
96.9	70	48	63
122	70	43	
144	77	53	52
164	77	87	
189	77	73	
219	67	50	32

**Table 5:** Measurement results for the 8.02 MHz ultrasonic transducer.

Sa µm	Gain dB	H_I_ %	H_II_ %
0.28	46	75	
26	46	74	
31.3	75	45	
74.3	87	49	
96.9	87	41	
122	90	41	
144	90	43	
164	90	30	
189	90	48	
219	90	36	39

## Data Availability

The raw data supporting the conclusions of this article will be made available by the authors on request.
